# Association between Respiratory Virus Infection and Development of *De Novo* Donor-Specific Antibody in Lung Transplant Recipients

**DOI:** 10.3390/v16101574

**Published:** 2024-10-05

**Authors:** Anoma Nellore, Julie Houp, John T. Killian, Ajit P. Limaye, Cynthia E. Fisher

**Affiliations:** 1 Division of Infectious Diseases, University of Alabama at Birmingham, Birmingham, AL 35294, USA; 2 Division of Transplant Surgery, University of Alabama at Birmingham, Birmingham, AL 35294, USA; 3 Division of Pathology, University of Alabama at Birmingham, Birmingham, AL 35294, USA; 4 Division of Infectious Diseases, University of California at San Francisco, San Francisco, CA 94143, USA; 5 Division of Infectious Diseases, University of Washington, Seattle, WA 98195, USA

**Keywords:** lung transplant, respiratory viral infection, HLA antibody, chronic lung allograft dysfunction, donor-specific antibody

## Abstract

Chronic lung allograft dysfunction (CLAD) is the most common cause of long-term lung allograft failure. Several factors, including respiratory virus infection (RVI), have been associated with CLAD development, but the underlying mechanisms of these associations are not well understood. We hypothesize that RVI in lung transplant recipients elicits the development of donor-specific antibodies (DSAs), thus providing a mechanistic link between RVI and CLAD development. To test this hypothesis, we retrospectively evaluated for the presence of HLA antibodies in a cohort of lung transplant recipients with symptomatic RVI within the first four months post-transplant using sera at two time points (at/directly after the transplant and following RVI) and time-matched controls without RVI (post-transplant). We found a trend toward the development of *de novo* DSAs in those with symptomatic RVI versus controls [6/21 (29%) vs. 1/21 (5%), respectively, *p* = 0.09]. No cases or controls had DSA at baseline. We also found increased rates of CLAD and death among those who developed class II DSA versus those who did not (CLAD: 5/7 (71.4%) vs. 19/34 (54.3%), death: 5/7 (71.4%) vs. 17/35 (48.6%)). Prospective studies evaluating the temporal development of DSA after RVI in lung transplant patients and the subsequent outcomes are warranted.

## 1. Introduction

While advances in surgical techniques, immunosuppression and pre-transplant HLA matching have improved one-year survival following lung transplant, long-term outcomes remain poor. The most common cause of lung graft failure and death after the first-year post-transplant is chronic lung allograft dysfunction (CLAD), which develops in approximately half of all lung transplant recipients (LTRs) by five years post-transplant [1]. The pathogenesis of CLAD is poorly understood, with multiple potential triggers, including T cell-mediated acute cellular rejection, chemical insult (e.g., reflux), and certain infections. Recent studies have also suggested that *de novo* HLA donor-specific antibodies (DSAs) may predict CLAD development [2]. HLA class II DQ, in particular, has been associated with the obstructive form of CLAD (bronchiolitis obliterans syndrome, BOS) [3]. Animal models of organ transplantation suggest that *de novo* DSAs may develop via pathologic activation of the innate and adaptive immune system after allograft injury [4]. Respiratory virus infections (RVIs) are common in LTRs, can cause direct acute allograft damage, and have also been associated with the development of CLAD in multiple studies [5,6,7,8,9,10,11,12,13,14,15,16,17]. However, these previous studies have been more correlative and did not address the potential mechanism(s) by which RVI may lead to CLAD, including the role of *de novo* DSAs in mediating chronic allograft damage after lung transplantation.

We hypothesize that RVI is linked to the development of pathologic *de novo* DSAs after lung transplantation, thereby providing a potential mechanism for the observed association of RVI with CLAD. To test this hypothesis, we retrospectively evaluated post-transplant *de novo* HLA antibodies from a biobank of longitudinal sera in a cohort of lung transplant recipients with either symptomatic RVI or matched controls without RVI and followed these patients long-term for development of CLAD and/or death.

## 2. Methods

### 2.1. Study Cohort and Design

We retrospectively identified 21 adult LTRs transplanted at the University of Washington between January 2007 and May 2012 who developed symptomatic RVI within the first 110 days post-transplant and who had serum available within 90 days prior to RVI (or at time of transplant) and within 6 months after RVI (cases). Cases were matched 1:1 to LTRs without symptomatic RVI during the same transplant time period (controls) based on the time of serum samples available post-transplant. Cases and controls were selected from a larger cohort of 250 LTRs, as outlined in the consort diagram (Supplementary Figure S1). To minimize bias, selection of cases and controls was based on the RVI status and sample availability only, and was performed blinded to clinical knowledge of patients, including pre-transplant HLA matching, DSA, and CLAD endpoints. Baseline demographic and transplant information were collected via electronic medical record review by trained personnel using standardized data collection forms. Some clinical information on the included patients has been previously published in a larger cohort study [5]; however, the prior study only examined the association between RVI and CLAD and did not examine DSA development. Furthermore, the current study is distinguished from prior work because it uses the updated 2019 consensus definitions for CLAD [18]. This study was approved by the University of Washington Institutional Review Board (IRB#44580).

### 2.2. Post-Transplant Follow-Up of LTRs

At the University of Washington, LTRs are followed closely for at least the first-year post-transplant, with outpatient visits occurring weekly for 4 weeks, every 2 weeks for 1 month, and every 2–3 months until 12 months post-transplant. Patients are instructed to perform home spirometry via a hand-held spirometer given to the patients at the time of their transplant, and they are instructed to tell their transplant team if there is a ≥10% decrease in the forced expiratory volume in one second (FEV1). Formal pulmonary function tests are performed with routine clinic visits and as clinically indicated. A decrease of ≥10% in FEV1 typically prompts investigation for underlying causes of this decline, including consideration for RVI testing if the patient has compatible symptoms. LTRs also typically have blood draws at least once a week for the first three months (i.e., for immunosuppression levels, CMV, PCRs, etc.); the sera used for this research study were obtained as part of a leftover sample biorepository. During the study period, approximately 262 lung transplants were performed at the University of Washington.

### 2.3. Respiratory Virus Testing

RVI testing was conducted only in patients who presented with upper and/or lower respiratory tract symptoms (e.g., fever, cough, rhinorrhea, coryza, sinus pain/pressure, sore throat, shortness of breath, etc.), had radiographic abnormalities, and/or had decreases in their spirometry. No surveillance testing in asymptomatic patients was performed. Since the decision to test for RVI was based on the clinician’s discretion and was not performed specifically for a research study, no standardized symptom surveys were used. Nasal swabs, washes, or BAL specimens were tested for respiratory viruses using either a direct fluorescent antibody and culture or a laboratory-developed PCR assay that tests for 12 viruses: respiratory syncytial virus (RSV), parainfluenza (PIV) 1–4, influenza A and B, adenovirus (ADV), coronavirus (CoV), rhinovirus (RHV), metapneumovirus (MPV), and bocavirus, as previously described [19,20,21,22,23,24]. Based on center protocols, BAL was performed with RVI testing if there was any concern for lower respiratory tract disease (i.e., abnormal chest imaging, more severe lower respiratory symptoms, such as shortness of breath, productive cough, or decrease in spirometry values). Given this testing algorithm, the RVI was considered lower tract if the respiratory virus was detected via BAL sample and upper tract if detected only by nasal swab or nasal wash.

### 2.4. Determination of CLAD

All available clinical, radiographic, and spirometry data were reviewed separately by two transplant pulmonologists who were blinded to the study results. In the case of disagreement in the diagnosis, the two reviewers conferred, and a consensus was reached in all instances. Per the 2019 International Society for Heart and Lung Transplant (ISHLT) Consensus Report, CLAD was defined as a decline in FEV1 to ≤80% of the patient’s baseline value for >3 months in the absence of clinical confounders [25]. The CLAD phenotype at CLAD onset was determined based on the available clinical data up to three months after CLAD diagnosis. Per the consensus statement, four types of CLAD phenotypes were considered: bronchiolitis obliterans syndrome [BOS], restrictive allograft syndrome [RAS], mixed, and undefined. The definitions and diagnostic criteria for these phenotypes are discussed in depth in the ISHLT Consensus Report [25]. Only the phenotype present at the diagnosis of CLAD was used as an endpoint in this study; evolution of CLAD phenotypes (e.g., from BOS to RAS) at later time points was not examined.

### 2.5. Laboratory Testing and Determination of DSA

Sera from cases and controls were evaluated for anti-HLA DSA on FlowPRA beads representing the HLA-A, -B, -Cw, -DR, -DQ, and -DP antigens. HLA testing on the research sera was performed using OneLambda kits (Thermo Fisher Scientific, Waltham, MA, USA). DSA that was not previously present and crossed the mean fluorescence intensity (MFI) threshold of >1500 was termed new or *de novo* DSA, as per standard guidelines [26]. DSA testing was conducted by personnel blinded to clinical status (case vs. control, CLAD vs. no CLAD).

### 2.6. Statistical Analysis

The primary endpoint was the percentage of LTRs among cases versus controls, who developed *de novo* DSA between the baseline and the follow-up time points. The exploratory endpoint was the association between the development of *de novo* DSA and a composite endpoint of CLAD and death. The chi-squared test or Fisher’s exact test (dichotomous) and Wilcoxon rank sum test (continuous) were used as applicable for the primary and exploratory endpoints and to compare baseline variables. Kaplan–Meier curves were also used to estimate and graph the probability of the composite endpoint of CLAD (including all sub-types) and death. Stata version 16 was used for all statistical analyses (StataCorp, College Station, TX, USA).

## 3. Results

### 3.1. Patient Cohort

Twenty-one LTRs with symptomatic RVI within the first 110 days post-transplant who also had sera available prior to and after the RVI episode were identified as cases, and twenty-one LTRs without symptomatic RVI and with similarly timed sera available were matched as controls (Supplementary Figure S1). Baseline demographic and transplant characteristics of the cases and controls are shown in Table 1; no statistically significant differences were seen. All patients underwent induction with Basiliximab per our center’s protocol. Maintenance immunosuppression included the use of a calcineurin inhibitor (predominantly tacrolimus), an antimetabolite (mycophenolic acid or mycophenolate mofetil), and prednisone. The major indications for transplant in this population were chronic obstructive pulmonary disease, interstitial pulmonary fibrosis, and cystic fibrosis. The median (interquartile range, IQR) among the transplant, the baseline, and the follow-up serum samples are shown in Table 1. Cases and controls were matched based on the number of days post-transplant when the serum samples were available for HLA testing, and the median (IQR) difference between the case and matched control samples was 1 day (IQR: 1–3 days) and 6 days (IQR: 2–8) for the baseline and follow-up serum samples, respectively. In cases, the median time from transplant to RVI was 71 (IQR: 50–88) days, the median time from the baseline sample was 54 (IQR: 15–69) days prior to RVI, and the median time from RVI to the follow-up serum sample was 131 (IQR: 90–153) days. The median (IQR) follow-up time from transplant to death or last follow-up was 3268 (IQR 2372–3917) days, with no statistically significant difference between cases and controls. Additionally, all cases and controls had full follow-up for CLAD or death through at least 1000 days post-transplant.

### 3.2. Details of Respiratory Virus Infection

The most common respiratory viruses in the RVI case cohort were seasonal CoV (n = 7, 31.8%) and RHV (n = 6, 27.3%), followed by PIV 1–4 (n = 4, 18.2%), RSV (n = 3, 13.6%), ADV (n = 3, 13.6%), influenza A (n = 1, 4.5%), and MPV (n = 1, 4.5%). One person had both CoV and RHV identified. Of the 21 cases, 19 (90.5%) had the virus identified on a lower respiratory sample (bronchoalveolar lavage) and 2 (9.5%) had the virus identified from a nasal wash sample.

### 3.3. RVI and Development of De Novo Donor-Specific Antibodies

Donor and recipient HLA typing was available for all subjects, and none had DSA detected at baseline. In 6/21 (29%) of the cases and 1/21 (5%) of the controls, *de novo* class II DSA was identified in the second sample (*p* = 0.09). All Class II DSA were identified at the DQ locus. No new class I DSA was identified among cases or controls. Post hoc analyses were performed on the majority of subjects (34 of 42) using HLA MatchMaker (www.epitopes.net) at the DQ locus; this revealed an average DQ difference of 1.43 in controls and 1.13 in cases, suggesting that cases and controls had similar degrees of HLA-DQ mismatch at baseline.

**Figure 1:**
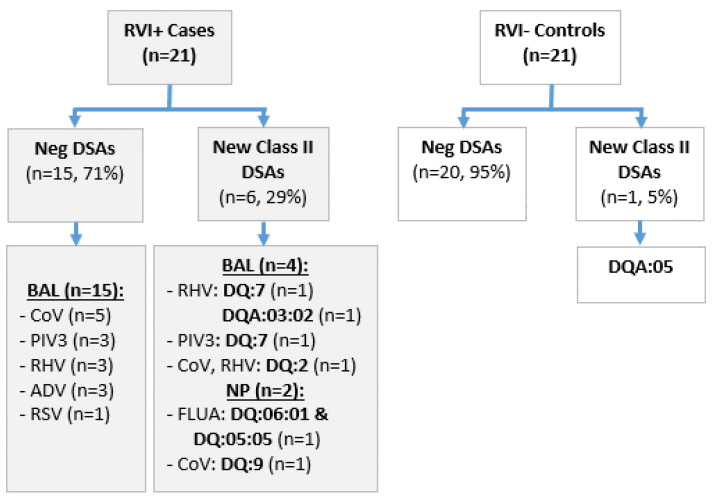
Flow chart depicts development of DSA in cases and controls and characteristics of RVI among cases. BAL, bronchoalveolar lavage. NP, nasopharyngeal swab. CoV, coronavirus (endemic). PIV3, parainfluenza virus 3. RHV, rhinovirus. ADV, adenovirus. RSV, respiratory syncytial virus.

**Figure 2:**
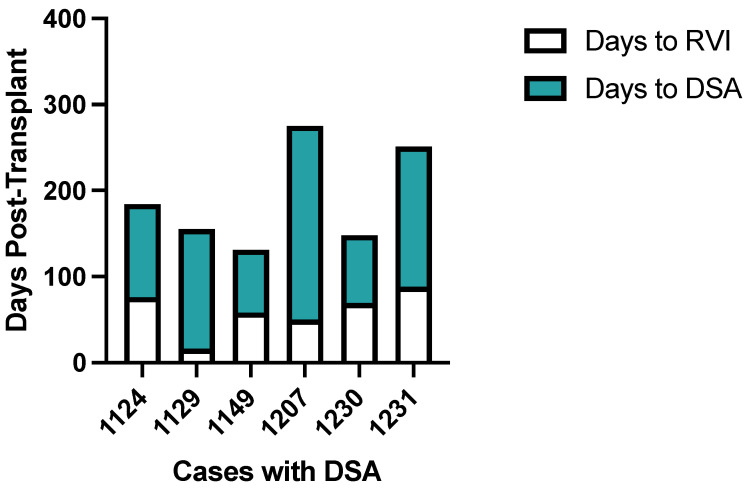
Bar plots show time (days) post-transplant for RVI and DSA development among the 6 cases under study. Days to RVI is indicated in white and days to DSA is indicated in aqua. De-identified case numbers are presented on the x-axis.

Figure 1 outlines the breakdown of the new class II DSAs in the cases by RVI type and location (BAL or nasal wash), and the new class II DSA in the control. Figure 2 demonstrates the time between RVI and DSA in six case subjects who developed DSA post-RVI. In these six subjects with *de novo* Class II DSAs, the median time to RVI was 64 days post-transplant (IQR: 50–76 days) and median time from RVI to DSA detection was 124 days after RVI (IQR: 79–163 days). None of the patients who had new DSA had preceding CMV pneumonia or pneumonitis. Similar proportions of patients, with and without DSA, had evidence of rejection prior to the second sample; of the seven patients who developed DSA, three out of seven (42.9%) had acute rejection diagnosed prior to the second sample (two of these were cases and the rejection were diagnosed either after the RVI or concurrently with the RVI, and one was the control). In those who did not develop DSA, 14/35 (40%) had rejection diagnosed prior to the second sample. 

### 3.4. DSA and Development of Chronic Lung Allograft Dysfunction and/or Death

**Figure 3:**
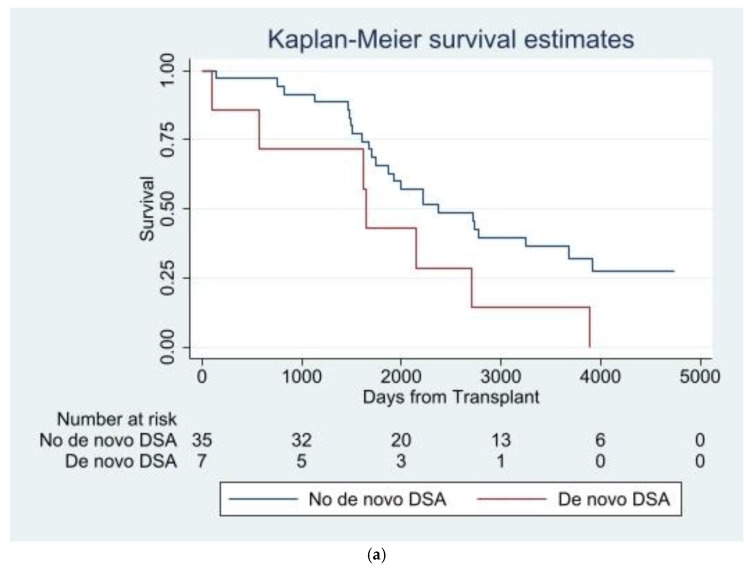

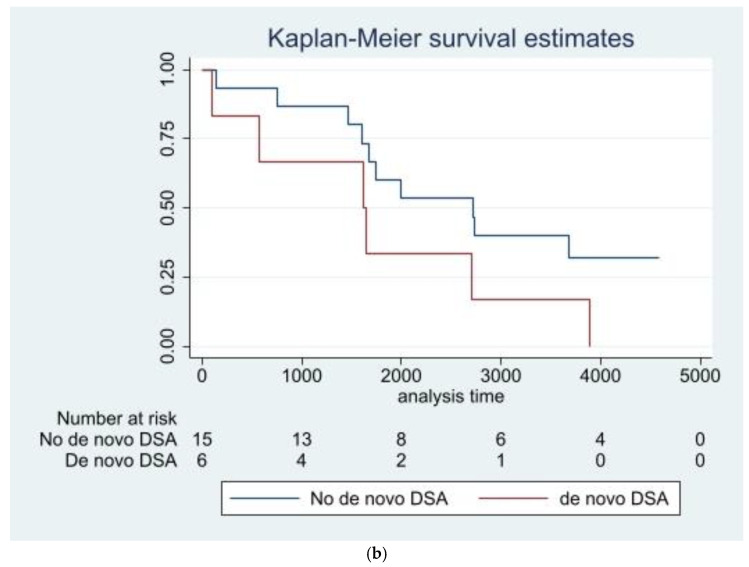
(**a**) Kaplan–Maier of composite endpoint of CLAD and death by development of *de novo* DSA. (**b**) Kaplan–Maier of composite endpoint of CLAD and death by development of *de novo* DSAs in LTRs with symptomatic respiratory virus infection (cases).

By the end of the follow-up period, 31/42 (73.8%) of the entire LTR cohort (cases and controls) had either developed CLAD (n = 24; 14 BOS, 1 RAS, 6 mixed, 3 undefined) or died prior to CLAD development (n = 7). Median (IQR) time to CLAD or death was 4.8 (4.1–7.4) years. Mortality at the end of the follow-up period (including those who had CLAD and subsequently died) was 22/42 (52.4%). Overall, 7/7 (100%) of the LTRs with *de novo* class II DSA (cases and controls) either developed CLAD or died: 5/7 (71.4%) developed CLAD, 5/7 (71.4%) died (including 3 patients who got CLAD first), and 2/7 (28.6%) died without CLAD. In contrast, among LTRs (cases and controls) who did not develop new class II DSAs, 24/35 (68.6%) either developed CLAD or died: 19/35 (54.3%) developed CLAD, 17/35 (48.6%) died (including 12 patients who got CLAD first), and 5/35 (14.3%) died without CLAD. Figure 3a shows a Kaplan–Meier curve for the composite endpoint of CLAD and death across the entire LTR cohort (cases and controls) who either developed new class II DSAs or did not. 

When the analysis was restricted to cases alone (only those with symptomatic RVI), 6/21 (28.6%) had *de novo* DSAs and 15/21 (71.4%) did not. CLAD developed in 14/21 (66.7%) of cases and 10/21 (47.6%) died (8 died after CLAD development). Five out of six (83.3%) cases with *de novo* class II DSAs vs. nine out of fifteen (60%) cases without *de novo* class II DSAs developed CLAD, 4/6 (66.7%) cases with *de novo* class II DSAs vs. 6/15 (40%) cases without *de novo* class II DSAs died, and 6/6 (100%) cases with *de novo* class II DSAs vs. 10/15 (66.7%) cases without *de novo* class II DSAs either developed CLAD or died (Figure 3b).

## 4. Discussion

In this study, we utilized a single-center cohort of lung transplant recipients with well-characterized respiratory viral infection and adjudicated CLAD to investigate the association between symptomatic RVI and the development of *de novo* DSA. As an exploratory analysis, we also described the long-term development of CLAD and death in LTRs who developed DSAs versus those who did not. We found that LTRs who developed *de novo* DSAs were those with documented prior symptomatic RVI at a frequency that approached statistical significance. Similar to the data reported from other cohorts of LTRs [13,14,15,16,17], we found that the development of *de novo* DSA, and in particular, HLA-DQ, ref. [3] was also associated with the onset of CLAD and death in our cohort.

Previous studies did not find an association between RVI and *de novo* DSA [27,28]. However, these studies used positive viral PCR alone without symptom assessment. Thus, we believe the discrepant findings between these studies and ours may be related to our use of symptomatic RVI as part of the inclusion criteria. We hypothesize that symptomatic versus asymptomatic RVI is more likely to be associated with tissue injury and a cytokine milieu conducive to the development of off-target alloimmune responses that may contribute to CLAD in the lung transplant recipient. For example, as has been described in autoimmunity [29], toll-like receptor and interferon-γ signals elaborated from the lung transplant recipient in response to viral infection may activate bystander pre-formed HLA-reactive memory B cells to differentiate into pathologic HLA-antibody secreting plasma cells. Alternatively, and similar to the off-target effects of viral infection on allo-active T cells, ref. [30] viral infection may induce the expansion of allo-active B cells that cross-react to both viral and HLA epitopes. These data raise the possibility of potential mechanistic linkages among symptomatic RVI, DSA development, and CLAD to be explored in future studies.

Our study has some limitations. First, due to the relatively small patient number in this single-center cohort, we had limited power to detect significant differences and to investigate possible confounders of the strong trend between symptomatic RVI and the development of DSAs. Although we were able to demonstrate similar proportions of acute rejection prior to the second sample in groups with *de novo* DSAs versus those without, we did not have comprehensive data on clinically relevant bacterial pneumonia preceding the development of the DSAs. Future prospective studies should focus on the systematic collection of these potential confounders. Second, we did not have uniform assessments of the RVI episodes, including symptom surveys, duration of viral shedding, or consistent imaging to assess the degree of lower tract disease. Third, we did not assess the development of cellular alloimmunity (e.g., alloreactive T cells) or non-HLA Abs after RVI as a contributing factor to CLAD. Fourth, we did not have allograft biopsies before and after RVI or before and after *de novo* DSAs to directly demonstrate allograft damage from either insult. Finally, our serum biobank was not collected at routine time points after RVI to more precisely define the temporal relationship between RVI and development of *de novo* DSAs.

Our study also had several strengths. We used a well-characterized cohort of LTRs with uniform and immediate post-transplant follow-up, included only symptomatic RVI cases, used newer definitions and an endpoint of adjudicated CLAD as previously described [31], and utilized a control group with closely matched timing of samples post-transplant. We also included an evaluation of cofounders from donor/recipient HLA mismatch and we found the majority of LTRs with (cases) or without (controls) symptomatic RVI did not have significant differences in HLA-DQ loci epitope matching as predicted by HLA MatchMaker (www.epitopes.net). Therefore, we do not believe that the development of *de novo* HLA-DQ DSAs in LTRs with prior symptomatic RVI is due to inherent differences in donor/recipient HLA matching between cases and controls.

Despite the above limitations, we believe that the hypothesis generating findings reported from this retrospective single center LTR cohort remain relevant today. For example, the majority of RVIs that we report here are from endemic coronaviruses with lower respiratory tract tropism. We know that LTRs have worse morbidity and mortality after SARS-CoV2 infection [32], but we do not yet have a complete understanding of the post-acute sequelae of SARS-CoV2 on the development of alloimmunity or long-term lung allograft dysfunction. Bystander tissue-restricted and autoimmune antibodies have been reported in the normal host after SARS-CoV2 infection [33,34,35], and these findings suggest that similar off-target immune responses may develop after SARS-CoV2 infection in LTRs [36,37]. Therefore, even though this cohort was enrolled prior to the SARS-CoV2 pandemic, these data document a trend toward off-target *de novo* DSA development after RVI from endemic coronaviruses and may possibly be relevant to long-term outcomes of LTR with SARS-CoV2 infection. 

In conclusion, the association between symptomatic RVI and CLAD has been reported by us and others [5,6,7,8,9,10,11,12,13,14,15,16,17]. Understanding the mechanisms mediating this association is necessary to develop strategies to prolong the life span of the lung allograft. While our data suggest the potential role of *de novo* DSAs in mediating CLAD development after symptomatic RVI, future prospective studies with larger numbers of LTRs and inclusive of LTRs with SARS-CoV2 are needed to confirm and extend these findings.

## Figures and Tables

**Table 1:** Baseline and sample characteristics in lung transplant recipients with and without RVI.

Characteristics	Cases, n = 21	Controls, n = 21
Age in years, median (IQR)	56 (51–64)	58 (45–63)
Female sex, n (%)	8 (38.1)	8 (38.1)
Underlying pulmonary disease, n (%)		
COPD/Bronchiectasis	8 (38.1)	6 (28.6)
IPF	6 (28.6)	6 (28.6)
Cystic Fibrosis	2 (9.5)	5 (23.8)
Other	5 (23.8) ^a^	4 (19.0) ^b^
Single lung transplant, n (%)	4 (19.0)	2 (9.5)
Year of transplant		
2007–2009	14 (66.7)	13 (61.9)
2010–2011	7 (33.3)	8 (38.1)
Days, transplant to first serum sample, median (IQR)	19 (15–23)	17 (13–21)
Days, transplant to second serum sample, median (IQR)	179 (154–225)	180 (154–225)

COPD: chronic obstructive pulmonary disease; IPF: idiopathic pulmonary fibrosis; ^a^ Alpha-1 antitrypsin (1), sarcoidosis (1), interstitial lung disease (3); ^b^ Alpha-1-antitrypsin (1), LAM (1), pulmonary hypertension (1), interstitial lung disease (1).

## Data Availability

The raw data supporting the conclusions of this article may be made available by the authors on request.

## Supplementary Materials

The following supporting information can be downloaded at: https://www.mdpi.com/article/10.3390/v16101574/s1.
