# Supplementary material for: Association between Respiratory Virus Infection and Development of De Novo Donor-Specific Antibody in Lung Transplant Recipients
Source: Viruses. 2024 Oct 5;16(10):1574. doi: 10.3390/v16101574 (PMC11512259; doi:10.3390/v16101574)
Supplement: Supplementary file 1 [file viruses-16-01574-s001.zip › viruses-3224229-supplementary.pdf]

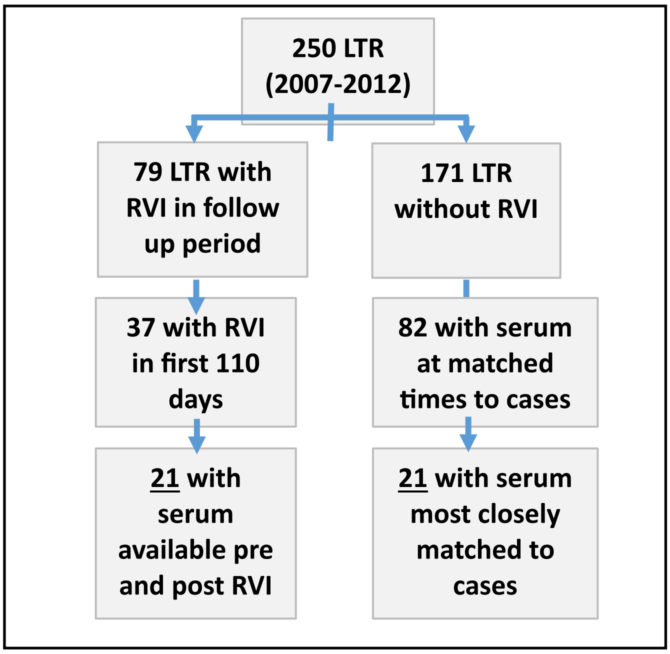

Supplemental Figure S1. Case-cohort extraction from parent cohort of LTR. Flow chart depicts how cases and controls were selected for this study.
